# Residual refinement for interactive skin lesion segmentation

**DOI:** 10.1186/s13326-021-00255-z

**Published:** 2021-12-18

**Authors:** Dalei Jiang, Yin Wang, Feng Zhou, Hongtao Ma, Wenting Zhang, Weijia Fang, Peng Zhao, Zhou Tong

**Affiliations:** 1 Echocardiography and Vascular Ultrasound Center, The First Affiliated Hospital, Zhejiang University School of Medicine, Hangzhou, CN China; 2 College of Computer Science and Technology, Zhejiang University, Hangzhou, CN China; 3 Department of Urology, The First Affiliated Hospital, Zhejiang University School of Medicine, Hangzhou, CN China; 4 Department of Medical Oncology, The First Affiliated Hospital, Zhejiang University School of Medicine, Hangzhou, CN China

**Keywords:** DCNN, Skin lesion, Interactive segmentation, Residual refinement, Two-stage pipeline

## Abstract

**Background:**

Image segmentation is a difficult and classic problem. It has a wide range of applications, one of which is skin lesion segmentation. Numerous researchers have made great efforts to tackle the problem, yet there is still no universal method in various application domains.

**Results:**

We propose a novel approach that combines a deep convolutional neural network with a grabcut-like user interaction to tackle the interactive skin lesion segmentation problem. Slightly deviating from grabcut user interaction, our method uses boxes and clicks. In addition, contrary to existing interactive segmentation algorithms that combine the initial segmentation task with the following refinement task, we explicitly separate these tasks by designing individual sub-networks. One network is SBox-Net, and the other is Click-Net. SBox-Net is a full-fledged segmentation network that is built upon a pre-trained, state-of-the-art segmentation model, while Click-Net is a simple yet powerful network that combines feature maps extracted from SBox-Net and user clicks to residually refine the mistakes made by SBox-Net. Extensive experiments on two public datasets, PH2 and ISIC, confirm the effectiveness of our approach.

**Conclusions:**

We present an interactive two-stage pipeline method for skin lesion segmentation, which was demonstrated to be effective in comprehensive experiments.

**Supplementary Information:**

The online version contains supplementary material available at 10.1186/s13326-021-00255-z.

## Background

After years of rapid growth, the DIKW (data, information, knowledge, and wisdom) hierarchy [1] has been closely related to the development of artificial intelligence and, more precisely, deep learning. As a paradigm of deep learning, AI algorithms hierarchically transform data into information, then knowledge, and finally wisdom by building deep layers of the network to represent different levels of abstraction. Artificial intelligence has contributed to the resolution of a variety of biomedical problems, including cancer and have the potential to deliver better management services to deal with chronic diseases. Nowadays, artificial intelligence methods have been progressively established as suitable tools for use in clinical daily practice. Deep learning is a subfield of artificial intelligence, which is highly flexible and have been applied in various areas of both basic and clinical research. One of the applications of deep learning that greatly benefits from this paradigm is image segmentation.

Image segmentation has an important role in medical diagnosis and research. Its results can help professionals to obtain accurate pathological regions, thus reducing the possibility of artificial empirical misjudgment. In its early days, experienced professionals worked assiduously to delineate diseased areas for better diagnosis. This kind of complete manual segmentation approach requires a significant amount of domain knowledge. Besides, it is time-consuming and laborious and could be severely affected by inter- and intra-observer variability [2]. In order to reduce the burden of manual segmentation, researchers have developed many automatic approaches [3–5] for medical image segmentation. These approaches range from early stage low-level-feature based methods such as Otsu [6], region growth [7], and grabcut [8] to deep convolutional neural network-based methods such as FCNs (fully convolutional networks) [9] and U-Net [10]. In medical image segmentation, U-Net is one of the most popular network architectures and has been commonly used in various medical imaging modalities [10, 11]. Fully automatic segmentation methods do not require user interaction, which greatly decreases the costs. However, this is a double-edged sword since there is no means for them to refine the segmentation result when it is not satisfactory. Thus, even the more sophisticated DCNN (deep convolutional neural network)-based methods could not achieve accurate and robust results that are clinically useful.

To address the limitations of automatic segmentation approaches, a trade-off was made between complete manual and fully automatic approaches. The interactive segmentation approach, which uses user interactions as input signals to guide segmentation, could alleviate the clinician’s burden and, at the same time, achieve satisfactory segmentation results by incremental refinement. ITK-SNAP [12] provides an interactive segmentation mechanism that employs an active contour model for segmentation that accepts user-provided seeds or blobs as a starting point. Although it is ‘Interactive’, it lacks the ability to refine the segmentation result, and its underpinning model is not powerful enough to model the variability of our target medical images. Similar approaches such as random walks [13], graph cuts [14] and grabcut [8] provide mechanisms to incrementally refine the segmentation result, but the performances of those methods are limited by the representativeness of the underpinning model since they only incorporate primitive low-level features for inference. Li [15] proposed a stacked adversarial learning (SAL) method based on an FCN to improve the dermoscopic image segmentation method. The authors build upon generative adversarial networks with a novel SAL architecture such that skin lesion features can be learned iteratively in a class-specific manner. However, the stability of the generated samples is not satisfactory; and complex parameter adjustment is required, which increases the time costs of the training model. Liu [16] proposed efficient skin lesion segmentation based on an improved U-net model, which mainly includes batch normalization and dilated convolution. However, the model regards dark regions as regions of interest, and the segmentation performance of the model was poor in a few cases where the region of interest was brighter than the surrounding skin region. Qin [17] proposed an asymmetric encode-decode network with two decoding paths for skin lesion segmentation. A skip pathway was designed to transfer the more representative features from the encoder to the decoder. However, in clinical work, the images are much more complex than the experimental data. Without the interaction of medical personnel, it is difficult to obtain a satisfactory segmentation result.

Due to the recent explosive growth of deep neural networks [18, 19] and their application in semantic segmentation problems [9, 10, 20, 21], the method for interactive object segmentation has experienced a swift change from traditional low-level-feature-based algorithms [13, 14, 22, 23] to deep convolutional neural networks [24–31]. Consequently, the results, in terms of accuracy and the intensity of user interaction, have improved tremendously.

**Fig. 1:**
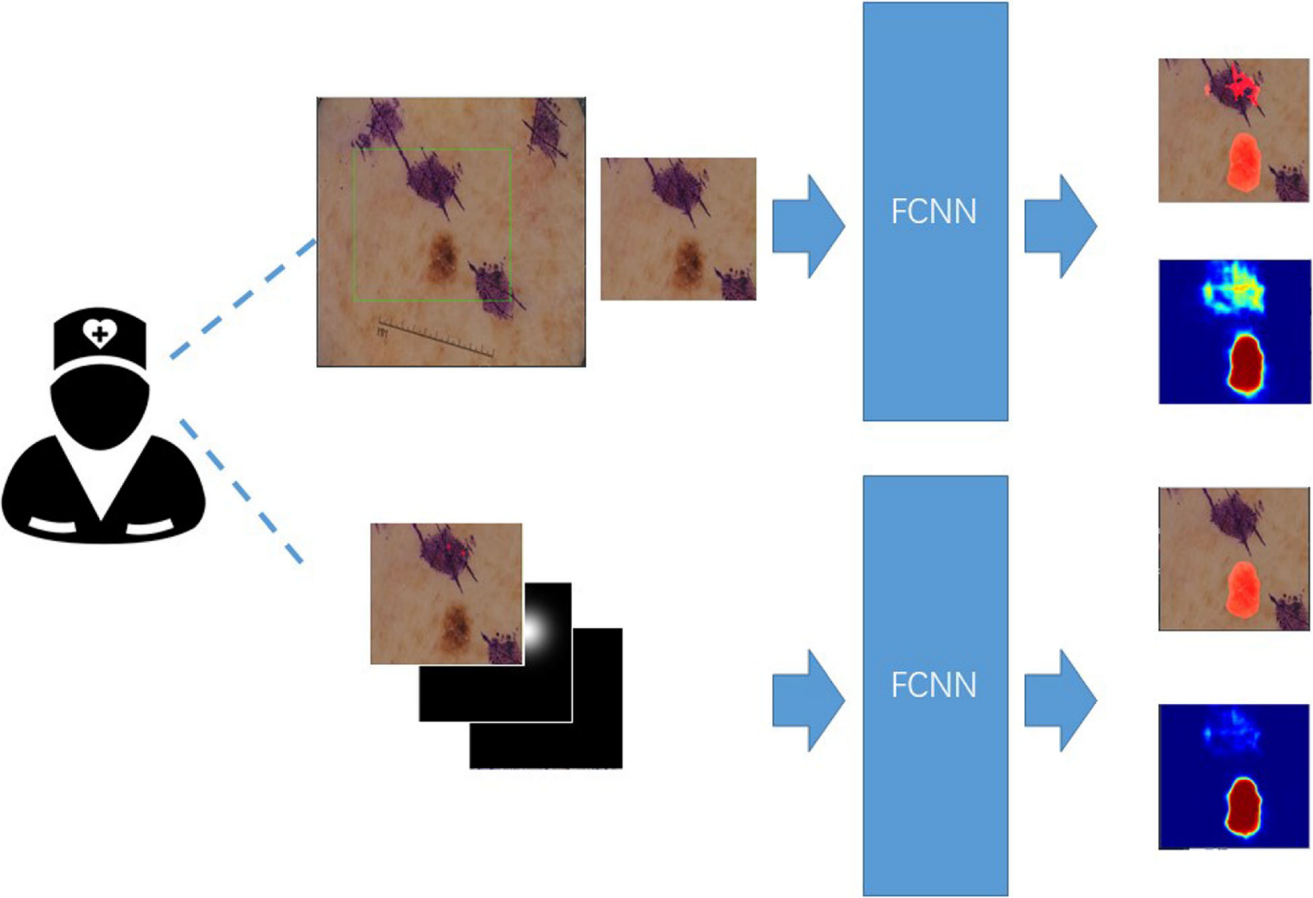
The workflow of our method. First, a box is drawn by the user who possesses domain knowledge to delineate the region of interest (ROI). The ROI is then cropped out to exclude most of the useless background and fed into a fully convolutional neural network to generate a segmentation. Most of the time, this segmentation will be very satisfactory. In addition, no further process is required. However, not all situations are ideal. Take the one in the figure as an example. There is a blot that we would like to remove. To address this type of situation, the user can provide another FCN with positive and negative clicks, which are transformed into Gaussian centred distance maps, to further refine the segmentation

In this article, in order to compensate for the defects of traditional methods that cannot effectively extract the deep information of images and because the depth model is not flexible enough for clinical use, we proposed a novel algorithm that combines the representational power of deep convolutional neural networks (DCNNs) and the flexibility of grab-cut (8)-like user interaction. While previous methods normally combine the initial segmentation task with the following refinement task in a single network, we explicitly separate them by designing individual sub-networks. One sub-network is SBox-Net, and the other sub-network Click-Net. Compared to the current skin lesion segmentation methods, our model has the characteristics of flexibility and precision. Clinicians can judge whether the segmentation results are satisfactory according to the segmentation model of the first-stage SBox-Net. If the segmentations are satisfactory, further refinement is not required. Otherwise, we can click on the pathological pictures. Click-Net will automatically process clinicians’ clicks and refine the segmentation results. Comprehensive experiments are then conducted to demonstrate the effectiveness of our approach. The workflow of our method is shown in Fig. 1.

## Methods

**Fig. 2:**
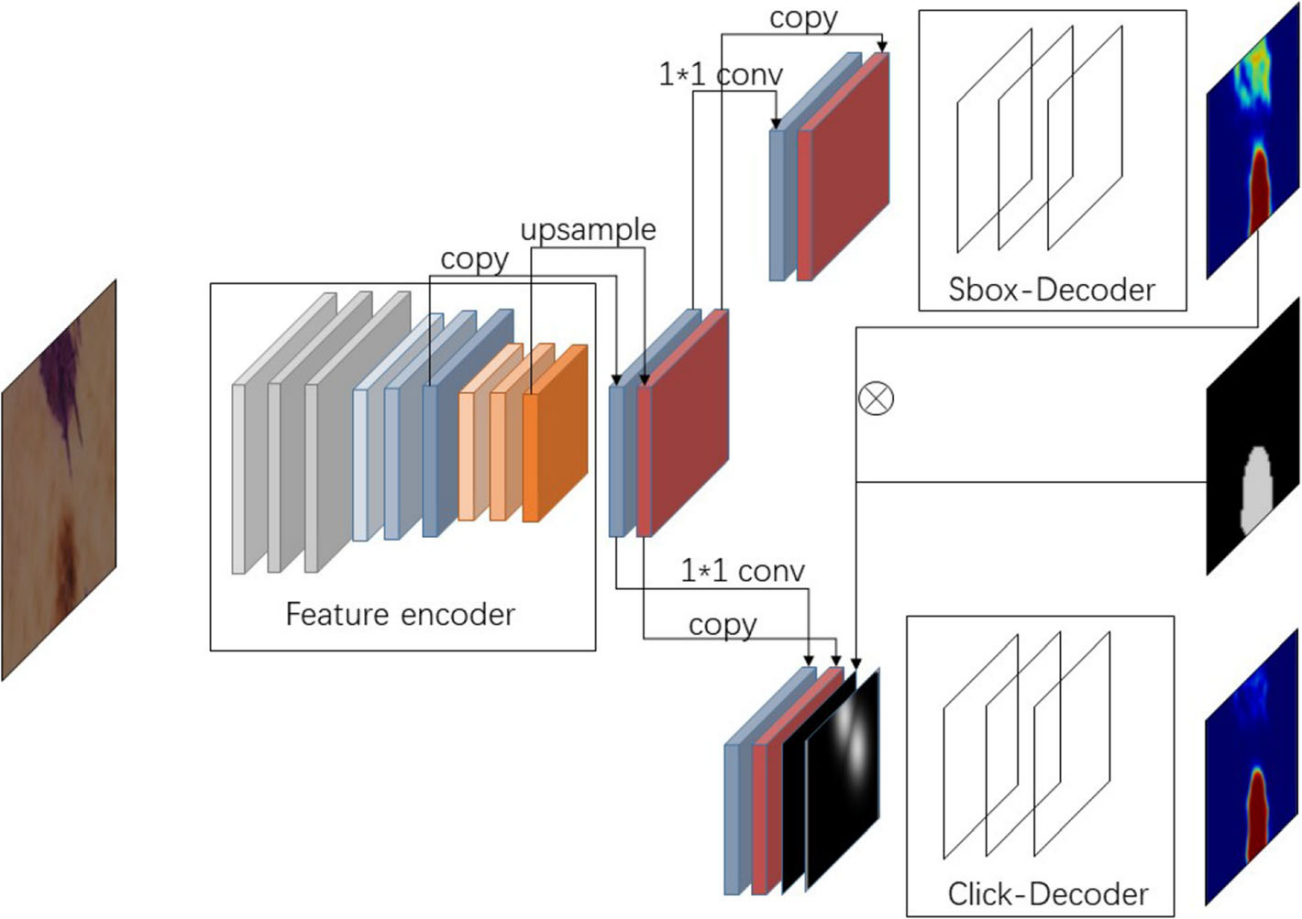
The overall architecture of our proposed method. It is composed of three major parts: a feature encoder for encoding features at different abstract levels, an SBox-Net for initial segmentation and a Click-Net for refinement. Using the feature encoder, we obtain feature maps at two levels of abstraction, namely, low-level features and high-level features. Our SBox-Net is used to predict segmentation at a coarse level; thus, we highlight our high-level features by reducing the number of channels of low-level feature maps. In addition, in our Click-Net, we reduce the channels of high-level features since our goal is to recover details according to user clicks. All previously mentioned channel reduction operations are performed by 1*1 convolution. Finally, we simulate user clicks by sampling from differences of SBox-Net segmentation and the ground truth (denoted by ⊗ in the figure)

The network structure consists of three main parts: 1. The network encoder, which was used to encode the features of different levels of abstraction. 2. SBox-Net. In addition to the feature encoder, we obtain features of two abstract levels, namely, low-level features and high-level features. SBox-Net highlights high-level features by reducing the number of channels of low-level feature mapping and obtains rough prediction segmentation. 3. Click-Net, whose main goal is to restore details according to the user’s clicks. We generated a Gaussian distance map with the same size as the input image by using the user’s clicks and used the Gaussian distance map as the weight of the segmentation results of the final upsampling layer of Click-Net. Finally, the segmentation results of Click-Net are refined according to the weight. The architecture of our proposed method is illustrated in Fig. 2.

### SBox-Net

Our SBox-Net was designed to be a binary segmentation network. Except for the last inference layer, there is no difference between our SBox-Net and a normal semantic segmentation network. Thus, SBox-Net could smoothly utilize a pre-trained state-of-the-art semantic segmentation network by simply replacing the top segmentation layer of an existing state-of-the-art model with our binary segmentation layer. We can then fine-tune the network to fit our goal. This strategy saves us considerable training time and computational resources. As for the simulation of a user drawing a surrounding box, we take the bounding box of the ground truth mask jittered randomly by up to 30 px in each direction. In this way, the randomness of user behaviour is well modelled.

As shown in Supplementary Fig. 1, in SBox-Net, in order to concatenate the shallow and deep features in the encoder, the features extracted from the encoder should be ‘concatenated’ first. Then, the 3 × 3 convolution is used to refine the features, and the refined features have deeper semantic features. During the upsampling process, factor = 4 bilinear interpolation is used to recover the pixel-wise prediction of the image resolution entered in the encoder. We define this prediction as a rough prediction; and in clinical segmentation, if the physician is satisfied with this result, he can obtain a satisfactory result without any manipulation. Otherwise, he can use Click-Net for refinement. Section “User interaction simulation” and “User interaction transformation” introduce some preliminary information about Click-Net, and section “Click-Net” describes Click-Net in detail.

### User interaction simulation

Surrounding box simulation is quite straight-forward, as previously stated in section “SBox-Net”. Click simulation requires slightly more caution.

Before delving into the details of click simulations, we need to go through the workflow of a typical interactive object segmentation process. First, a user draws a surrounding box around the target object. Based on the surrounding box, the SBox-Net will perform one pass of inference on the patch of the image cropped by the surrounding box. If the result needs to be refined, typically, it would contain two types of mistakes, namely, extra pixels and left-behind pixels (from a user’s perspective). In these two types of mistakes, a user adds clicks to refine the segmentation result.

By separating our architecture into SBox-Net and Click-Net, we can perfectly simulate those two types of mistakes during training time. After a forward pass of SBox-Net, we obtain a preliminary result. We then can calculate the differences between the preliminary result and the ground truth mask, obtaining the false positives and false negatives of the preliminary result, which are a close simulation of the two types of mistakes previously mentioned. Thus, we can directly sample clicks on false positives and false negatives (see Fig. 2). Our strategy for simulating user clicks is simpler, more straightforward and more effective than that introduced by [24].

### User interaction transformation

At the inference time of our Click-Net, a user can provide positive and negative clicks to refine the results of SBox-Net. All user interactions can be grouped into two sets: a positive click set S^1^, which contains all user-proved positive clicks; and a negative click set S^2^, which contains all user-provided negative clicks. A Gaussian distance transformation was used to transform those two sets into two separate channels G^1^ and G^2^, respectively. Both G^1^ and G^2^ were initialized to zero. Let ${G}_{\left(m,n\right)}^1$ and ${G}_{\left(m,n\right)}^2$ be the elements at location (m, n) in matrices G^1^ and G^2^, respectively, which are calculated by:

1 $$ {G}_{\left(m,n\right)}^1=\underset{s_{i,j}\in {S}^1}{\max }{e}^{-\frac{4\ast \left({\left(m-i\right)}^2+{\left(n-j\right)}^2\right)}{R^2}} $$

2 $$ {G}_{\left(m,n\right)}^2=\underset{s_{i,j}\in {S}^2}{\max }{e}^{-\frac{4\ast \left({\left(m-i\right)}^2+{\left(n-j\right)}^2\right)}{R^2}} $$

where R is a radius parameter that controls the area of influence of a user click. After the transformation of user clicks, we concatenate the feature maps extracted from SBox-Net with G^1^ and G^2^, which are then fed into Click-Net for further processing.

### Click-Net

The workflow of Click-Net is shown in Supplementary Fig. 2. On the basis of SBox-Net segmentation, our Click-Net was designed specifically for responding to user clicks when a user is seeking to refine the segmentation result. In order to achieve this, the training data for Click-Net must be collected carefully. The click simulation strategy is described in detail in section “User interaction simulation”. In Click-Net, we first transform the positive and negative clicks into two Gaussian centred maps. We then concatenate the transformed Gaussian maps with feature maps extracted from SBox-Net, which are then fed into Click-Net to generate our final segmentation. Contrary to previous works [24–26], we do not concatenate the transformed user clicks with raw images directly but with feature maps instead. The main motivation behind this is to decouple the segmentation process and the refinement process. Besides, it is obvious that user clicks are informative both semantically (positive or negative) and spatially (the absolute position of the clicks inside the surrounding box). Thus, their level of abstraction is more compatible with high-level features instead of low-level features such as raw pixels.

Inspired by the famous ResNet [19], which incorporates residual blocks to tackle the exploding gradient problem and significantly boosts the performance of artificial networks, we designed our Click-Net as a residual refinement network. Before yielding the final segmentation, our Click-Net fuses its output with that of the SBox-Net, which makes it in effect a residual refinement network. The fusion process considers the number and position of user clicks. We transform the user clicks into a weight map using Gaussian distance transformation. Unlike in user interaction transformation depicted in 2.3, we do not differentiate between positive and negative clicks. Besides, instead of setting the pixel value to the maximum Gaussian distance from all click points, we add those distances up. Finally, the radius parameter, R, which controls the area of influence of a user click, is set to a much larger value, allowing each click to adjust the weight of a much broader area. The final weight map is given as:

3 $$ {W}_{\left(m,n\right)}=\sum \limits_{s_{i,j}\in \left({S}^1\cup {S}^2\right)}{e}^{-\frac{4\ast \left({\left(m-i\right)}^2+{\left(n-j\right)}^2\right)}{R^2}} $$

In Formula 3, *W*_(*m*, *n*)_ represents the sum of the Gaussian distances between all the click points and the element at location (m, n) in matrix W. ${e}^{-\frac{4\ast \left({\left(m-i\right)}^2+{\left(n-j\right)}^2\right)}{R^2}}$ represents the Gaussian distance from a single click point *s*_*i*, *j*_ in the set *S*^1^ ∪ *S*^2^ to the element at location (m, n) in matrix W.

After obtaining the weight map, we can fuse the SBox-Net result, denoted B, with the Click-Net result, denoted C, to produce our final result, denoted F, using the formula:

4 $$ F=W\ast C+B $$

where ∗ is the bitwise multiplication operator and + is the bitwise addition operator.

## Results

### Datasets

Our method has been trained and evaluated on two publicly available datasets, the ISIC dataset [32] and PH2 [33]. The ISIC dataset was used for a skin image analysis challenge hosted by the International Skin Imaging Collaboration (ISIC). The challenge was hosted in 2018 at the Medical Image Computing and Computer Assisted Intervention (MICCAI) conference in Granada, Spain. The dataset included over 12,500 images across 3 tasks, including lesion segmentation, attribute detection, and disease classification. To train and evaluate our interactive segmentation method, we selected the dataset for lesion segmentation, which consists of 2596 skin lesion images with corresponding segmentation masks. We divide the dataset into two independent and equally distributed parts: one part for training and the other part for model evaluation.

The other dataset, PH2, was provided by a joint collaboration between the University of Porto and University of Lisbon in conjunction with the Department of Dermatology at the Pedro Hispanio Hospital in Matosinos, Portugal. The dataset was mainly created to provide a common dataset that may be used for the performance evaluation of different computer-aided dermoscopic image diagnosis systems. The dataset consists of 200 dermoscopic images with corresponding segmentations, including 80 common nevi, 80 atypical nevi, and 40 malignant melanomas. All images are 8-bit RGB colour images with a resolution of 768*560 px. All dermoscopic images were carefully acquired using a magnification of 20* under unchanged conditions.

### Training configuration

Our SBox-Net utilizes the DeepLab V3+ model pre-trained on the Pascal VOC dataset with the last inference layer replaced. We then fine-tune our model using stochastic gradient descent with a batch size of 8 objects for 50 epochs. The learning rate is set to 0.01 with a momentum of 0.9 and a weight decay of 0.0005. Inspied by [34], we exploit the ‘poly’ learning rate policy, which multiplies the learning rate by (1 − *iter*/ max  _ *iter*)^*power*^ .

We trained our Click-Net with a learning rate of 0.1, while the other hyper-parameters remained the same. The objects we used to train Click-Net are those that are not accurately segmented by SBox-Net (IoU less than 0.9). We simulate user clicks by randomly sampling the false positives and false negatives of the SBox-Net prediction.

The experiments are conducted using the PyTorch framework. All our networks are trained on a single NVIDIA GeForce GTX TITAN X GPU with 12 GB of memory. The training of SBox-Net takes approximately 11 h, and Click-Net takes 6 h. The hyper-parameters of Sbox-Net and Click-Net are shown in Supplementary Table 1. We trained our Click-Net with a learning rate of 0.1 while the other hyper-parameters remained the same.

### Performance evaluation metrics

Our proposed method is composed of two loosely coupled modules with Click-Net using the feature maps extracted from SBox-Net, while our SBox-Net works fairly well without the knowledge of Click-Net. We evaluate our method in two stages. In the first stage, the performance of SBox-Net is evaluated. Then, we will show how our Click-Net improves the segmentation result of SBox-Net. The following performance metrics were used in evaluating our algorithm: the sensitivity (Sen), specificity (Spe), dice coefficient (Dic), accuracy (Acc) and intersection over union (IoU). The sensitivity, also known as the true positive rate, is the number of correctly segmented lesion pixels, and the specificity is the ratio of correctly segmented non-lesion pixels. The Dice coefficient evaluates the similarity between the segmented lesions and the underlying ground truth. The accuracy shows the overall pixel-wise segmentation performance. Finally, IoU, as its name implies, measures the proportion of the intersection over the union between the segmentation and the ground truth. All aforementioned evaluation metrics are calculated by the following formulas:

5 $$ Acc=\frac{TP+ TN}{TP+ FN+ TN+F\mathrm{P}} $$

6 $$ Sen=\frac{TP}{TP+ FN} $$

7 $$ Spe=\frac{TN}{TN+ FP} $$

8 $$ Dic=\frac{2\ast TP}{2\ast TP+ FN+ FP} $$

9 $$ IoU=\frac{Area\ of\ intersection}{Area\ of\ union} $$

### Segmentation performance

**Fig. 3:**
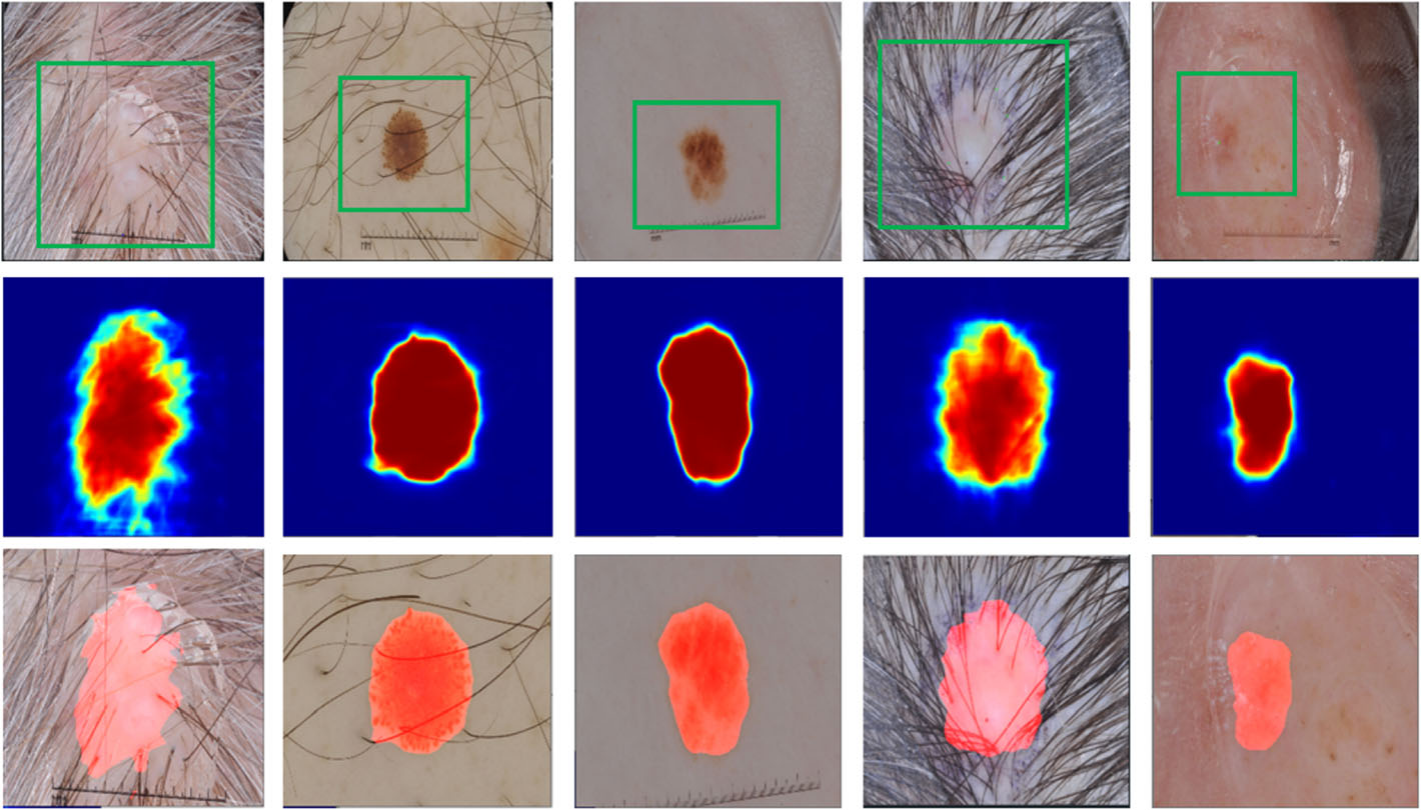
Example segmentations produced by our SBox-Net. The first row presents the original skin lesion images. The green boxes in those images are surrounding boxes provided by users that serve as guiding signals for SBox-Net. The middle row lists the segmentation confidence map generated by SBox-Net for the region of interest cropped by the surrounding box. The third row shows cropped images with corresponding overlayed segmentations

We demonstrate our algorithm on two publicly available datasets, ISIC 2018 and PH2. First, we present the performance of SBox-Net on those two datasets as it is the first stage of our proposed pipeline. Table 1 summarizes the segmentation performance of SBox-Net. From this table, it is obvious that all the evaluation metrics results on the ISIC dataset are higher than those on the PH2 dataset. The major reason is that the ISIC dataset is larger than the PH2 dataset. As a result, the model with more training data has better performance. As we can see, the proposed model achieves compelling results on both datasets, reaching an accuracy, a sensitivity, a specificity, a Dice coefficient, and an intersection over union of 94.40, 94.27, 91.60, 91.60, and 88.22% on PH2, respectively; compared to 96.23, 97.58, 92.52%, 92.93, and 90.89% on ISIC, respectively. Additionally, Fig. 3 illustrates some examples of the segmentation results of our SBox-Net.

**Table 1:** Skin lesion segmentation performance of SBox-Net

Datasets	Acc (%)	Sen (%)	Spe (%)	Dic (%)	IoU (%)
PH2	94.40	94.27	91.60	91.60	88.22
ISIC	96.23	97.58	92.52	92.93	90.89

In Table 2, the performance of stacking a Click-Net on top of SBox-Net is listed. The performance improvement is significant considering the relatively small amount of computation required by the light-weight Click-Net. The table shows that the improvement made on the PH2 dataset is larger than that on the ISIC dataset. This is partly because our SBox-Net already did a very good job on the ISIC dataset (reaching a 90% IoU). There is less room left for improvement. Conversely, the PH2 dataset provides a good place for our Click-Net to shine.

**Table 2:** Skin lesion segmentation performance of SBox-Net + Click-Net with the number of clicks restricted to 2. The numbers in parentheses are the relative improvements made by adding a Click-Net

Datasets	Acc (%)	Sen (%)	Spe (%)	Dic (%)	IoU (%)
PH2	95.53(+ 1.13)	96.34(+ 2.07)	93.82(+ 2.22)	93.12(+ 1.52)	90.36(+ 2.14)
ISIC	96.86(+ 0.63)	98.48(+ 0.90)	92.63(+ 0.11)	94.06(+ 1.13)	92.31(+ 1.42)

**Fig. 4:**
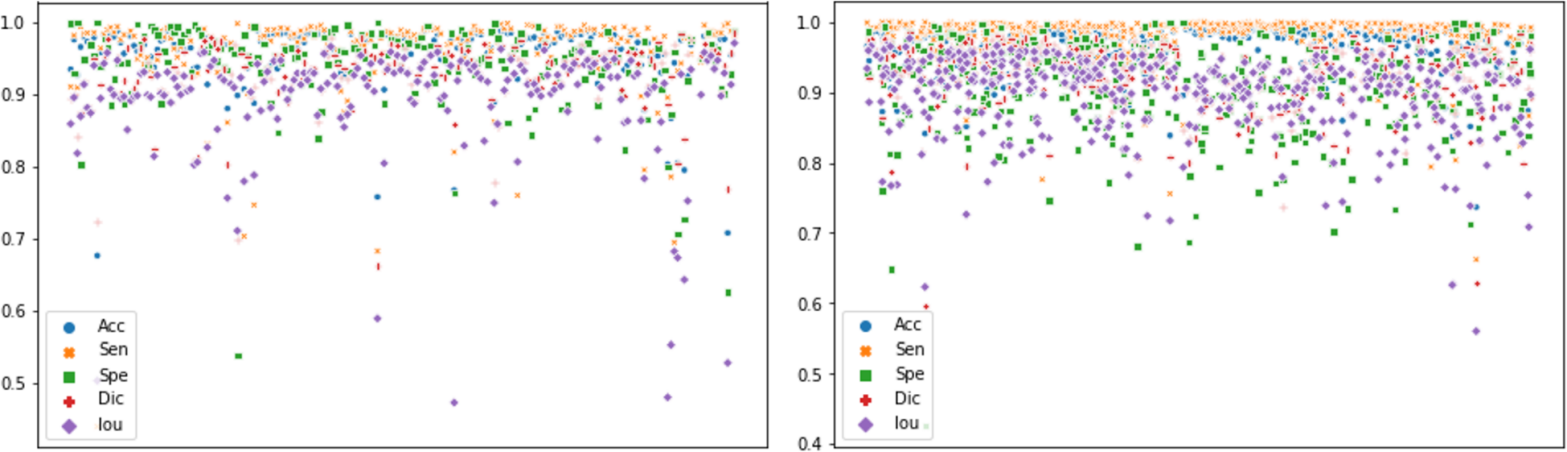
The distribution of performance metrics on both datasets. We can see from the scatter plot that apart from a few outliers, our method achieved very good scores on most of the samples, concentrated at approximately 0.9 and above

In Fig. 4, the overall performance of our method on each single image instance of both datasets is depicted as scatter plots. These plots show that our method achieved very good scores on most of the samples in those two datasets. In the figure, the horizontal axis represents the index for each sample, and the vertical axis represents the evaluation metric score. Some outliers with metric scores lower than 0.7 can be spotted. Each of these outliers corresponds to a sample, and these samples are difficult to segment because the labelling is not precise or the sample is inherently hard.

### Interactive performance

In this subsection, we present the performance of our algorithm in terms of user interactions. In Table 3, both the number of clicks and estimated time required to achieve a certain IoU on the two datasets are listed. Since our SBox-Net requires at least 2 clicks to draw a surrounding box, we fix the amount of user interaction of our SBox-Net to 2 clicks. Drawing a surrounding box is an easy task that takes less than 1 s on average. Identifying an ill-segmented area and then clicking requires more attention. Our simulation experiments showed that this process takes 1.4 s on average. In the evaluation process, we adopted the same sampling strategy as depicted in section “User interaction simulation”.

**Table 3:** Number of clicks and estimated time to achieve a certain IoU on 2 datasets

Datasets	IoU (%)	Clicks	Time (seconds)
PH2	90	3.67	3.4
	95	6.72	7.6
ISIC	90	2	< 1
	95	4.84	4.9

### Residual refinement performance

**Fig. 5:**
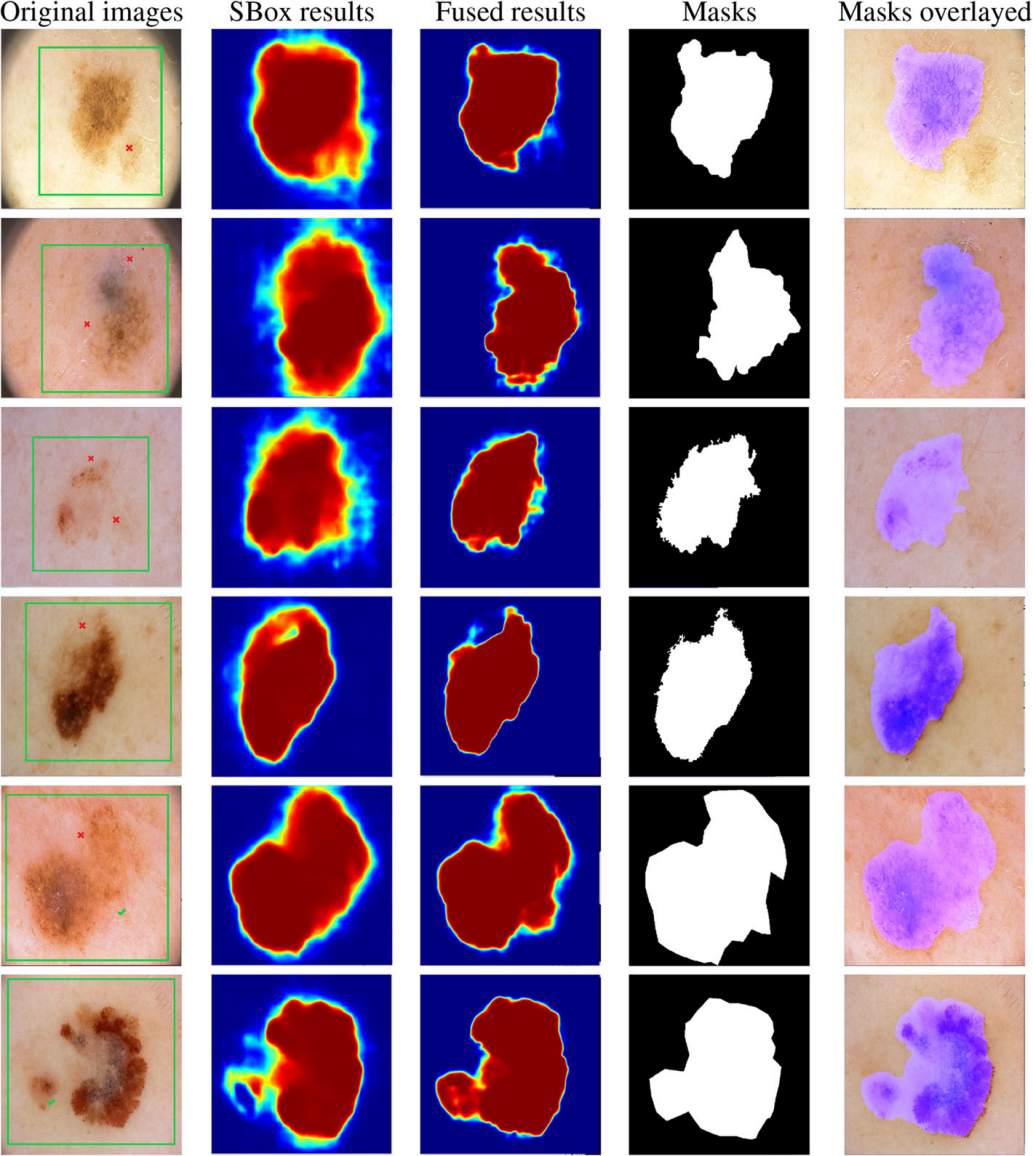
Residual refinement process of our proposed method. In the rare situation of our SBox-Net producing unsatisfactory segmentation results, our Click-Net can be invoked to refine the results. First, a surrounding box (the green box in column a) is drawn by the user to select the region of the target lesion. Based on the surrounding box, a preliminary result is returned by SBox-Net. While the results are not satisfactory, the user can provide positive clicks (the green check marks in column a) and negative clicks (the red cross marks in column a) to further refine the segmentations. The third column shows the fused segmentation maps of Click-Net and SBox-Net compared to the SBox-Net results and ground truth masks in the second and fourth columns, respectively. In the final column, the fused segmentations overlayed on the cropped original images are presented

In Fig. 5, we illustrate our residual refinement process. After a user draws a surrounding box around the target lesion region, our SBox-Net produces an initial segmentation. In cases where the initial segmentation is unsatisfactory, Click-Net will be invoked. After each click a user inputs, Click-Net transforms the clicks into Gaussian distance maps. Concatenated with feature maps extracted from SBox-Net, the newly formed input is then fed into Click-Net to generate its immediate output. Before yielding the final segmentation, our Click-Net first reevaluates its output by multiplying the weight map (see “Click-Net”) pixel-wise. The output is then added to that of SBox-Net, reaching the final segmentation. Note that the process can be repeated to refine the result step by step. A few examples are illustrated in Fig. 5.

### Ablation study

Global Context Extractor: As a common practice in recent deep segmentation methods, various types of global context extractors have been proven to be very useful. The most successful ones are Atrous Spatial Pyramid Pooling (ASPP) and Pyramid Scene Parsing (PSP). We conducted experiments to evaluate the effectiveness of ASPP and PSP in our interactive segmentation task. As a comparison, we have also experimented with a naive convolution layer as our global context extractor. The results of our experiments are shown in Table 4. As the table shows, if we only use a naive convolution layer as our global context extractor, its performance only reaches an MIoU of 83.23%. We add the PSP module to obtain more abundant global features and significantly improved segmentation results. It is important to note that ASPP is superior to PSP in the experimental results, and ASPP achieves the optimal performance, reaching an MIoU of 88.22% with SBox-Net alone and an MIoU of 90.36% when combined with Click-Net.

**Table 4:** Comparison of different global context extractors

Global Context Extractor	PSP	ASPP	Naive
SBox IoU (%)	0.8632	0.8822	0.8323
SBox + Click IoU (%)	0.8942	0.9036	0.8532

## Discussion

### Related works

Image Segmentation: For the first time, an FCN [9] adopted convolutional neural networks (CNNs) for dense prediction by replacing fully connected layers with convolutional layers. This innovation enables the FCN to process different sized input images and produce segmentation maps accordingly. Almost all the subsequent state-of-the-art approaches for segmentation followed this paradigm. To address the resolution loss due to pooling layers in CNNs, two types of architectures have emerged. The first one is the encoder-decoder architecture. U-Net [10] is one of the representatives of this class. The encoder module gradually reduces the spatial resolution with pooling layers while the decoder recovers the object details and spatial dimension using shortcut connections. Another branch of architectures drops pooling layers altogether and instead uses a special type of convolution layer, called dilated/atrous convolution. Representatives of this class include DeepLab [35] and PSPNet [36].

Interactive Segmentation: Various types of user interaction have been exploited for interactive segmentation. Xu et al. [24] proposed a method to incorporate user clicks into an FCN [9] model by transforming user clicks into Euclidean distance maps, which are then concatenated with the RGB channel of the original image and fed into the iFCN model. This paradigm is followed by subsequent interactive segmentation architecture. A follow-up model by Liew et al., called RIS-Net [26], improved the result of Xu et al. [24] by focusing on local regions that are poorly segmented. RIS-Net exploits the local regional context around the user click pair along with multiscale global contextual information to improve the segmentation result. DEXTR [25], a more recent method proposed by Maninis et al., uses four extreme points on the object as a supervisory signal to guide the segmentation. Slightly different from previous methods, DEXTR encodes these points as a Gaussian map. DeepIGeoS [37] strictly followed the iFCN paradigm but transformed user-provided clicks and scribles into a geodesic distance map instead of a Euclidean distance map for interactive medical image segmentation. In the research of Wang et al. [38], a method for image-specific fine-tuning at test time for a CNN model was proposed.

To segment an object in an image, a box around the object, which we called the surrounding box, is first drawn by users. Unlike a tight, accurate bounding box, the surrounding box is much looser and thus a user-friendlier version of the bounding box. The surrounding box is a user-provided guiding signal to exclude most of the background from the image. After feeding the patch of the image cropped by the surrounding box into SBox-Net, an initial segmentation result is directly returned. If the result is satisfactory, no more user interaction is required. Otherwise, users provide positive and negative clicks, which are then transformed into Gaussian-centred maps. Those maps together with feature maps extracted from SBox-Net are then fed into Click-Net for further refinement. Through careful architectural design, our method requires only one feed-forward pass through SBox-Net while Click-Net reuses the feature maps computed by SBox-Net in the following refinement iterations. Another key improvement that separates our method from the existing methods is that our Click-Net is designed specifically for responding to user clicks when a user seeks to refine an unsatisfactory result. We achieve this by applying a simple, straightforward, yet effective sampling strategy in the training process of Click-Net. User intentions are well captured by our Click-Net. Apart from addressing the issues stated above, the modulization design of our architecture brings us several advantages. First, since SBox-net operates directly on raw images, instead of (image, transformed user interaction) pairs as in [24–26], our SBox-Net can smoothly repurpose pre-trained, state-of-the-art semantic segmentation models for our task by simply replacing the inference layer. This strategy saves us a huge amount of training time and computational resources. Second, as SBox-net and Click-Net are only loosely coupled, we could conveniently test different Click-Nets in a plug-and-play style. For instance, we could test an aggressive Click-Net at some time and a moderate Click-Net at others. Alternatively, to push the limit even further, we could test a totally different refinement network with another type of user interaction, such as scribbles. Our architecture, therefore, can be seen more broadly as a flexible framework for interactive skin lesion segmentation and can be readily extended to accommodate various types of user interactions. However, this paper focuses only on clicks, leaving others for future research.

In summary, the key contributions of this paper are summarized as follows:

- We combined a deep convolutional neural network with a grabcut-like user interaction to tackle the interactive skin lesion segmentation problem.
- We decoupled the refinement task with the initial segmentation task using the modularized design of the network architecture, which greatly enhances the flexibility of our model, facilitates reusing computations at inference time and allows our Click-Net to be trained in a way that fully captures the intention of users.
- We exploit the pre-trained, state-of-the-art semantic segmentation model for SBox-Net. With few changes in the model and a small training time budget, we could achieve a compelling result.

## Conclusion

In this paper, we present an interactive method for skin lesion segmentation. We approach the problem as a two-stage pipeline. First, a user uses a surrounding box to select the skin lesion of interest. A preliminary segmentation result is returned by our SBox-Net. Then, if the result in the first step is unsatisfactory, a light-weight Click-Net is invoked to further refine the segmentation. Extensive experiments on two public datasets, PH2 and ISIC, proved the effectiveness of our approach.

## Supplementary Information

**Additional file 1 **: **Supplementary Figure 1.** The workflow of Sbox-Net. In SBox-Net, in order to integrate the shallow and deep features in the encoder, the features extracted from the encoder should be ‘concatenated’ first. Then, the 3 × 3 convolution is used to refine the features, and the refined features have deeper semantic features.

**Additional file 2 **: **Supplementary Figure 2.** The workflow of Click-Net. In Click-Net, we first transform the positive and negative clicks into two Gaussian centred maps. We then concatenate the transformed Gaussian maps with the feature maps extracted from SBox-Net, which are then fed into Click-Net to generate our final segmentation.

**Additional file 3 **: **Supplementary Table 1.** Hyper-parameters of SBox-Net and Click-Net.

## Data Availability

All the data can be downloaded at https://challenge2018.isic-archive.com.

## Abbreviations

ROI: Region of interest
CNNs: Convolutional neural networks
ISIC: International Skin Imaging Collaboration
ASPP: Atrous Spatial Pyramid Pooling
PSP: Pyramid Scene Parsing
FCN: Fully convolutional networks
DCNN: Deep convolutional neural network
SAL: Stacked adversarial learning
