# Supplementary figures and images for: Residual refinement for interactive skin lesion segmentation
Source: J Biomed Semantics. 2021 Dec 18;12:22. doi: 10.1186/s13326-021-00255-z (PMC8684232; doi:10.1186/s13326-021-00255-z)

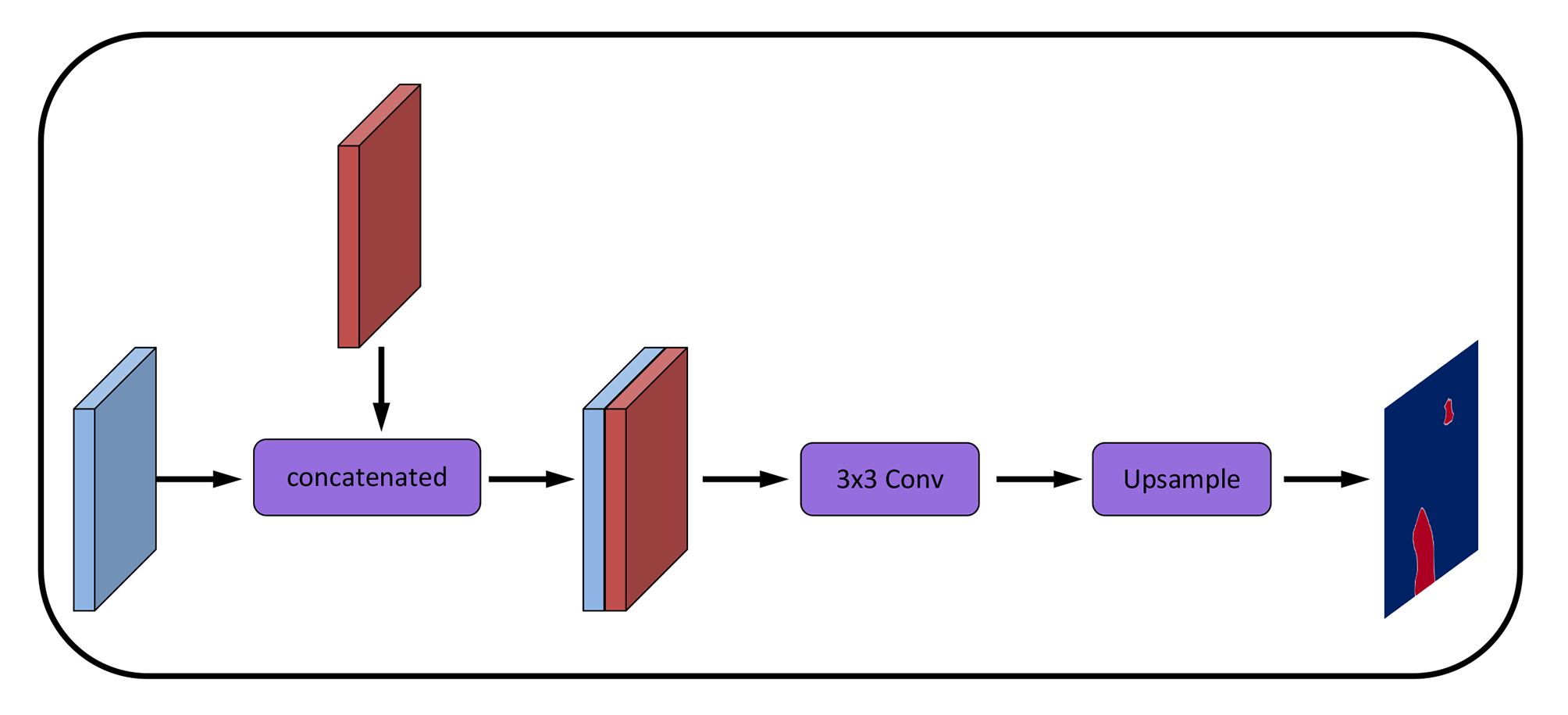

Supplement: Supplementary file 1 — Additional file 1 : Supplementary Figure 1. The workflow of Sbox-Net. In SBox-Net, in order to integrate the shallow and deep features in the encoder, the features extracted from the encoder should be ‘concatenated’ first. Then, the 3 × 3 convolution is used to refine the features, and the refined features have deeper semantic features. [file 13326_2021_255_MOESM1_ESM.tif]

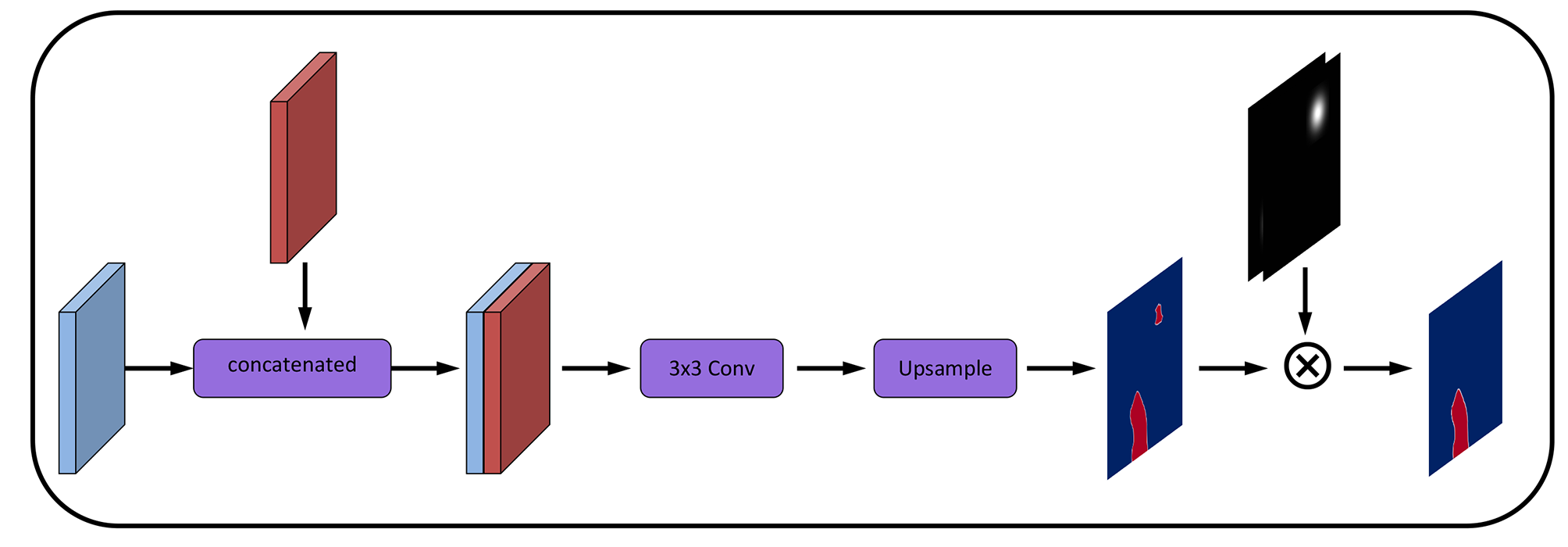

Supplement: Supplementary file 2 — Additional file 2 : Supplementary Figure 2. The workflow of Click-Net. In Click-Net, we first transform the positive and negative clicks into two Gaussian centred maps. We then concatenate the transformed Gaussian maps with the feature maps extracted from SBox-Net, which are then fed into Click-Net to generate our final segmentation. [file 13326_2021_255_MOESM2_ESM.tif]
