# Supplementary material for: Residual refinement for interactive skin lesion segmentation
Source: J Biomed Semantics. 2021 Dec 18;12:22. doi: 10.1186/s13326-021-00255-z (PMC8684232; doi:10.1186/s13326-021-00255-z)
Supplement: Supplementary file 3 — Additional file 3 : Supplementary Table 1. Hyper-parameters of SBox-Net and Click-Net. [file 13326_2021_255_MOESM3_ESM.docx]

**Supplementary Table 1** Hyper-parameters of SBox-Net and Click-Net

| Sbox-Net, **Click-Net** | Hyper-parameter |
| --- | --- |
| *Optimizer*  *Batch size*  *Epochs*  *Learning rate*  *Momentum*  *Weight decay*  *Warm up* | stochastic gradient descent (SGD)  8  50  0.01, **0.1**  0.9  0.0005  poly |
